# Supplementary material for: Mental Well-Being and Sexual Intimacy among Men and Gender Diverse People Who Have Sex with Men during the First UK COVID-19 Lockdown: A Mixed-Methods Study
Source: Int J Environ Res Public Health. 2022 Jun 7;19(12):6985. doi: 10.3390/ijerph19126985 (PMC9222884; doi:10.3390/ijerph19126985)
Supplement: Supplementary file 1 [file ijerph-19-06985-s001.zip › Supplementary File S1.pdf]

Supplementary File S1 Items and measurement

Age group was aggregated from continuous variable responses to 'What is your age in years?' to the categories: 16-24, 25-34, 35-44, 45+ years. A variable combining relationship status and living circumstances was generated from the relationship status variable and the living circumstances variable. Relationship status was measured with the item 'What is your relationship status? (Single/ In a monogamous relationship/ In an open relationship/ It's complicated/ Other). Living circumstances was measured with the item 'How many people are in lockdown with you at your address?' (No-one, I am alone at the moment/ Parents and/or other family/Romantic partner(s)/Regular house or flat mates/Children/Other). The Short Warwick-Edinburgh Mental Well-being Scale was used to capture mental well-being comprising seven items that each pertain to respondent feelings and thoughts over the previous six weeks and relate respectively to: feeling optimistic, relaxed, useful, dealing with problems well, thinking clearly, feeling close to others, and being able to make decisions (each item carrying five response options that indicate frequency). Total scores were calculated using the recommended additive approach and these were then dichotomized into 'possible or probable depression' ≤20 or 'average to or high well-being' >20 . Number of casual partners was measured with the item 'Please indicate the approximate number of casual sex partners you have met for sex since social distancing began' (open response). Change in number of casual partners was measured with the item 'Has the number of your casual sex dates with one person changed since social distancing measures began' (Reduced a lot/Reduced a bit/Stayed the same/Increased a bit/Increased a lot). A similarly worded item with the same response options asked about sex dates 'involving three or more people e.g. threesomes or group sex'. Non-physical sexual contact was measured using the item 'Has the number of occasions you had sex without physical contact (e.g. webcam sex, phone sex, sexting, exchange of naked pictures) changed since social distancing measures began in March 2020?' (Reduced a lot/Reduced a bit/Stayed the same/Increased a bit/Increased a lot). Sexual network app use was measured with the item 'Has the number of occasions you opened a sexual networking app (eg Grindr, Scruff, Tindr, or any other) changed since social distancing measures began in March 2020? (Reduced a lot/Reduced a bit/Stayed the same/Increased a bit/Increased a lot). Change in time spent just chatting online was measured by asking 'Has the amount of time you spend just chatting to people on apps (without intending to arrange a hook-up) changed since social distancing measures began in March 2020?' (Reduced a lot/Reduced a bit/Stayed the same/Increased a bit/Increased a lot). Pandemic anxiety was measured with LIKERT scale responses to the question 'How much of the time do you feel anxious about the coronavirus outbreak overall?'using a six-point LIKERT scale for response options ranging from 'None of the time' to 'All of the time'.
