# Supplementary material for: Mental Well-Being and Sexual Intimacy among Men and Gender Diverse People Who Have Sex with Men during the First UK COVID-19 Lockdown: A Mixed-Methods Study
Source: Int J Environ Res Public Health. 2022 Jun 7;19(12):6985. doi: 10.3390/ijerph19126985 (PMC9222884; doi:10.3390/ijerph19126985)
Supplement: Supplementary file 1 [file ijerph-19-06985-s001.zip › Supplementary File S2.pdf]

Supplementary FileS2: topic guide

The use of sexual networking apps during the coronavirus (Covid-19) outbreak (2020) among gay, bisexual and other men who have sex with men: a mixed-method study

Interview Schedule.

Personal situation

What's your current relationship situation? Do you have a main or regular partner(s)?

What's your living situation like during this lock-down? How's that working out for you?

Have you been sharing a home since the coronavirus outbreak started and/or since lock-down started in the UK (on 24th of March)?

(If in a relationship) Does your partner live with you now?

Do you ever use sexual networking apps like *Grindr, Scruff, Tinder, Recon* etc?
Which of these apps do you have profiles on?

Impact of Coronavirus

How are you feeling about the coronavirus outbreak? Has it affected your life much? In what ways? (Anxiety levels?)

Are you able to go to work?
Impact on main relationships?

What do you know about coronavirus transmission and sex?

Emotions

How satisfied are you with the amount of physical intimacy that you are getting since the coronavirus outbreak and/or since lock-down?
Have your needs for physical contact been satisfied?

Apps / sexual behaviour

How do you generally use sexual networking apps (e.g. *Grindr, or another app)*?

Has your use of sexual networking apps changed in any way since the coronavirus outbreak?

Would you say you are using the apps more or less frequently?
What you use them for?

Can you tell me what impact (if any) lockdown had on your sexual behaviour?

Did this change over time or was it constant?

How did you think about risk during this period? Sexual? Related to COVID-19?

Can you describe what actions, if any, you've taken to reduce risk during this period?

Has lockdown changed the places where you have sex? What about sex in saunas / cruising etc.

If you were to meet someone for sex now, would you be more likely to go to their place or yours? Why is that?

Can you describe to me your feelings about the limitations on sex that lockdown did and has posed? What have they meant to you?

Is it possible for men to carry on limiting the amount of casual sex they have? Until the coronavirus risk ends?

Interactions with another gay/bi men

Do you think the lockdown and the crisis in general will change the ways gay/bi men's relate it each other? Temporarily and/or longer-term?

Getting to know each other?

Steady fuck-buddies?

Relationships?

Accessing services

Have you ever had an HIV diagnosis?

(*If yes*) Has the coronavirus crisis had any impact on your regular HIV care? Can you describe what has happened?

Do you feel you know enough about how coronavirus affects people with HIV? (Promt: what would you want to know that you don't?)

(*if no*) Have you been using PrEP for HIV prevention ever? Since January 2020? How would you usually take your PrEP? (daily vs event based)

Have you had any communication from your PrEP clinic since the coronavirus outbreak?

How do you feel about that?

Did you change your PrEP use during lockdown? Can you tell me about your decision making process?

What impact, if any, had lockdown had on your access to HIV & STI (**delete as appropriate**) testing services?

Have you used any sexual health testing services since the coronavirus outbreak began?

(*if* yes)

How have you accessed these services? (prompt: was this different than

before?)
Have you had any difficulties?

(*if no*)
Was accessing these services something that you wated to do but couldn't?
Can you describe the difficulties you faced?
